# Bacterial Profile, Antibacterial Resistance Pattern, and Associated Factors from Women Attending Postnatal Health Service at University of Gondar Teaching Hospital, Northwest Ethiopia

**DOI:** 10.1155/2018/3165391

**Published:** 2018-02-25

**Authors:** Abebaw Bitew Kifilie, Mulat Dagnew, Birhanemeskel Tegenie, Biruk Yeshitela, Rawleigh Howe, Ebba Abate

**Affiliations:** ^1^Department of Medical Laboratory Science, College of Medicine and Health Sciences, Debre Markos University, Debre Markos, Ethiopia; ^2^Department of Medical Microbiology, School of Biomedical and Laboratory Science, College of Medicine and Health Science, University of Gondar, Gondar, Ethiopia; ^3^Armauer Hansen Research Institute, Addis Ababa, Ethiopia; ^4^Department of Immunology and Molecular Biology, School of Biomedical and Laboratory Science, College of Medicine and Health Science, University of Gondar, Gondar, Ethiopia

## Abstract

**Introduction:**

Surgical site infection is a vital cause of maternal mortality and morbidity, especially in resource-limited countries. The rise of antibiotic resistance bacterial infection poses a big threat to this vulnerable population. However, there is lack of studies around the study area.

**Objective:**

The purpose of this study was to identify bacterial profile, antibacterial resistance pattern, and associated factors among mothers attending postnatal care health service.

**Methods:**

Institutional based cross-sectional study was conducted on 107 study participants at University of Gondar Teaching Hospital from 1 January 2016 to 30 May 2016. Wound swab, aspirate, and biopsy were collected and performed for culture and drug resistance testing. Data were entered and analyzed by using SPSS version 20. Bivariate and multivariate logistic regression models were fitted to determine the associated factors for bacterial infection. Odds ratio (95% CI) was calculated to determine the strength of statistically significant associated factors.

**Result:**

Bacterial growth was confirmed in 90 (84.1%) of 107 study participants suspected to have surgical site infection. The predominant bacterial isolates were* S. aureus* (41.6%),* E. coli* (19.8%),* K. pneumoniae* (13.9%),* coagulase negative Staphylococcus* (12.9%), and* Enterobacter* spp. (4%). The majority of isolates were resistant to ampicillin, amoxicillin, and tetracycline but susceptible to ceftriaxone and amikacin. Multidrug-resistant bacteria species were isolated. Using a procedure such as cesarean section and episiotomy for delivery and premature rapture of membrane had strong association with bacterial infection.

**Conclusion:**

The high prevalence of bacterial profile and isolation of multidrug-resistant bacteria pose a big threat to postnatal mothers and their children. Factors such as cesarean section, episiotomy for delivery, and premature rapture of membrane were predictors for bacterial infection. Therefore, there should be done a continuous surveillance as well as rational use of antibiotics and a longitudinal study using phenotypic and genotypic methods will be done.

## 1. Introduction

Surgical site infection (SSI) is an infection that occurs on the skin and subcutaneous tissue within 30 days of surgical incision or deep tissue surgical procedure [1]. It is characterized by the basic sign of redness, pain, swelling, raised incision tissue temperature, and systemic fever [2, 3]. It also causes infection to women pelvic organs when normal flora of the women's genital and gastrointestinal tract contaminates the sterile amniotic fluid [4]. Women who give birth by cesarean section delivery have 5-fold to 20-fold risk of bacterial infection than women who give birth vaginally [5]. The predominant pathogenic bacteria isolated from infected wound include* Escherichia coli (E. coli)*,* Streptococcus* species,* Enterococcus faecalis* (*E. faecalis*),* Staphylococcus aureus (S. aureus),* Coagulase negative* Staphylococcus* (CoNS),* Gardnerella vaginalis*, and genital mycoplasmas [6, 7]. Bacteria that are isolated from SSI are too common to resist multiple classes of antibiotics. The downfall of efficient antibacterials will weaken our capacity to manage contagious disease in vulnerable patients undergoing cesarean section delivery [8]. World health leaders have declared that antibiotic-resistant bacteria are hurtful bacteria that cause a devastating risk to people in the world [9].

Generally bacterial infections during cesarean section delivery are among the principal sources of maternal mortality, which accounts for nearly one-tenth of global maternal death [10]. Annually the global estimate of SSIs is from 0.5% to 15%; this leads to an estimate of 358,000 maternal deaths in the world, of which 99% were in developing countries and half of which were in Sub-Saharan Africa; besides death, women who are exposed to peripartum infections are at risk of severe morbidity and long-term disabilities such as chronic pelvic pain, fallopian tube blockage, secondary infertility, and prematurity of the child [11, 12]. The risk of SSI was associated with premature rupture of membranes, prolonged labour, malnutrition, diabetes mellitus, obesity, lack of preincision antimicrobial care, which increases patients hospital stay, readmission, cost of care, and mortality [13, 14]. Decreasing the number of deliveries by C/S and identifying associated factors for SSI could contribute to decreasing maternal morbidity [7, 15].

Screening and treating bacterial infection during postnatal periods can improve future pregnancy and delivery outcomes. However, as far as our knowledge is concerned, there is a lack of studies conducted on bacterial profile and antibiotic susceptibility pattern among women attending postnatal service in developing countries including Ethiopia; as a result, the magnitude is not well known and is not comprehensive. To confirm proper treatment, recent information about organism, source of maternal infection, and its drug sensitivity test is mandatory. The information compiled here will increase the awareness of significant bacterial infection during postnatal periods, so periodic evaluation of bacterial profile and its drug response is needed to bring updated information. Therefore, this study determines bacterial profile, antibacterial susceptibility pattern, and associated factors among women attending postnatal health services.

## 2. Materials and Methods

### 2.1. Study Design, Area, and Period

An institutional based cross-sectional study was conducted at University of Gondar Teaching Hospital from January 2016 to May 2016. Gondar town is located 738 km far away from Addis Ababa, the capital city of Ethiopia.

### 2.2. Sample Size and Sampling Technique

A total of 107 women who have an infection due to cesarean section (CS) and episiotomy delivery were self-reporting to the hospital and were sampled for laboratory culture confirmation during the study period.

### 2.3. Sociodemographic Data and Specimen Collection

Sociodemographic variables such as age, residence, marital status, educational level, and occupation and other relevant clinical data such as gravidity, parity, and mode of delivery were collected using structured questionnaire. The specimen was collected by trained midwife nurses after getting ethical clearance from the School of Biomedical and Laboratory Science ethical review committee and informed written consent from study participants. Each biological specimen was collected for bacteriological laboratory analysis such as culture, Gram stain, biochemical test, and antibacterial susceptibility test.

### 2.4. Wound Specimen Collection and Laboratory Processing

The area around surgical and episiotomy site was cleaned with normal saline in order to reduce contamination. Then 107 wound specimens were collected by wound swabbing, Levine technique, and Z-technique by using sterile cotton tipped applicator stick, aspirating by needle and cutting wound biopsy depending on the wound type. Wound exudates were collected prior to wound cleaning during swabbing, but in Levine technique and Z-technique wound exudates were collected after wound cleaning.

Wound swabbing using Levine technique involves the rotation of swab over 1 cm^2^ area with sufficient pressure to fast fluid release from wound, whereas Z-technique requires the rotation of swab between 2 fingers by zigzag motion from margin to margin of wound at 10 points [16, 17].

Cotton tipped applicator stick that contains specimen was socked to a test tube containing Tryptone Soya broth in order to prevent drying and transported to microbiology laboratory.

The sample was inoculated onto blood agar plate (BAP) (Oxoid), MacConkey agar (MaC) (Oxoid), and Mannitol Salt Agar (MSA) (Oxoid) and incubated aerobically for 18–24 hours at 37°C. Identification of bacteria was performed based on colony morphology such as size, pigment, and edge with the naked eye from culture media and Gram stain. Gram stain was used to distinguish Gram-positive and Gram-negative isolates. Biochemical characteristics such as catalase, coagulase, bacitracin, novobiocin, and optochin were used to identify Gram-positive pathogenic bacterial species, whereas triple sugar iron agar, indole test, motility test, urea test, hydrogen sulfide production, citrate test, and lysine decarboxylase test results were used to identify Gram-negative pathogenic bacterial species.

### 2.5. Antibacterial Susceptibility Test

Antibacterial susceptibility testing was done by using modified Kirby-Bauer disk diffusion method and interpreted according to Clinical and Laboratory Standard Institute (CLSI, 2016) guidelines [18]. Around 3–5 pure colonies of bacteria were taken and transferred to a tube containing 5 ml sterile nutrient broth (Oxoid) and mixed gently until a homogenous suspension was formed and incubated for 3–5 hours until the turbidity of the suspension becomes adjusted to the density of 0.5% McFarland standards. Then they were inoculated to Müller-Hinton agar (MHA, PH = 7.2–7.4) (Oxoid) by using sterile swab evenly with 60°. The inoculums were allowed to dry for 5–15 minutes. Antibacterial discs were distributed 15 mm away from the edge and ≥24 mm apart from each other.

Antibiotic discs penicillin (10 IU), cloxacillin (30 *μ*g), tetracycline (30 *μ*g), clindamycin (2 *μ*g), gentamycin (10 *μ*g), nalidixic acid (30 *μ*g), ciprofloxacin (5 *μ*g), amikacin (30 *μ*g) ampicillin (10 *μ*g), amoxicillin (10 *μ*g), cefoxitin (30 *μ*g), cefixime (5 *μ*g), ceftriaxone (30 *μ*g), trimethoprim sulfamethoxazole (1.25/23.75 *μ*g), and ceftazidime (30 *μ*g) were used for SSI. Then the plates were incubated at 37°C for 24 hours and the results were interpreted according to the most recent version of Clinical Laboratory Standard Institute (CLSI 2016) [18]. The criteria to select the antimicrobial agents were based on availability, CLSI guide line, the organisms' Gram reaction, and frequent prescription of drugs for the management of postnatal infections.

### 2.6. Data Management, Analysis, and Presentation

The collected data were coded and transferred from a questionnaire to a computer file. Data were entered and statistically analyzed by SPSS version 20 software. Descriptive statistics were used to analyze frequency, mean, range, and standard deviation of sociodemographic characteristics and obstetric and clinical variables of study. Tables and figures were used to present the findings.

Bivariate and multivariate logistic regression models were fit to determine possible associated factors with bacterial infection. Odds ratio was used as a measure of the strength of association and reported with 95% confidence intervals (95% CI) to determine statistically significant association of risk factors with bacterial infection. *p* value ≤ 0.05 was considered to be statistically significant.

### 2.7. Data Quality Control

All steps in data collection and recording were monitored. The reagents were checked for expiry date and appropriate storage of temperature and humidity. SOPs were prepared and strictly followed. The quality of culture media and antimicrobial susceptibility testing was checked by using quality control standard strains of* E. coli *ATCC 25922,* S. aureus *ATCC 25923,* E*.* faecalis *ATCC 29212, and* K. pneumoniae* ATCC^@^BAA1705. 0.5% McFarland standards were used to standardize the inoculum density of bacterial suspension for susceptibility test. The acceptance range of 0.5% McFarland optical density is 0.08–0.1 [18].

### 2.8. Ethical Consideration

Ethical clearance was obtained from University of Gondar, School of Biomedical and Laboratory Sciences (SBMLS) ethical review committee for the initiation of the study. There was no additional sample to be taken from the study participants but only for the sake of this study. A written informed consent was obtained from mothers after explaining the purpose and objective of the study to them. Participants would have full right to continue or withdraw from the study. All information was kept confidential by assigning code and assessed only by principal investigator. The laboratory results were communicated timely with physicians and nurses for better patient management.

## 3. Result

### 3.1. Sociodemographic, Obstetric, and Clinical Variables

A total of 107 women with symptoms of infections during postnatal periods were investigated in this study. The mean age of the study participants was 26.21 (±5.5) and ranged from 15 to 44. The majority of the participants were urban dwellers (77.6%), orthodox Christian (90.7%), married (98.1%), and housewives (60.7%). A total of 107 postnatal clinic (PNC) participants were delivered from health institution, 52 (48.6%) of them had history of prolonged labour, 43 (40.2%) had history of premature rapture of membrane, and 81 (48.5%) were delivered cesarean section. Most of those participants had a history of 1–3 gravida (88.8%) (Table 1).

### 3.2. Isolation of Pathogenic Bacteria from SSI

**Figure 1:**
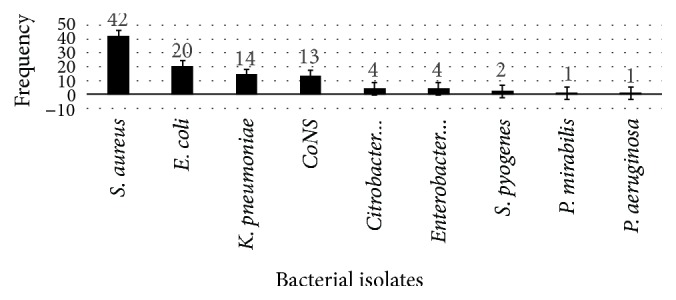
Profile and frequency of bacterial isolates from women having SSI during postnatal period at the University of Gondar Teaching Hospital from 1 January 2016 to 30 May 2016.

Of all the study participants, 107 wound specimens were collected based on physician request at the University of Gondar Teaching Hospital. From these suspected clinical specimens, the overall pathogenic bacterial infections were 90 (84.1%). Among these, 79 (87.8%) infections were due to single bacterial isolates, whereas 11 (12.2%) infections were due to mixed bacterial isolates. So a total of 101 bacterial isolates were isolated. Majority of them were Gram-positive and the predominant were* S. aureus*, 42/101 (41.6%), and CoNS, 13/101 (12.9%), followed by Gram-negative* E. coli*, 20/101 (19.8%), and* K. pneumoniae*, 14/101 (13.9%) (Figure 1).

### 3.3. Antibacterial Susceptibility Pattern for Bacterial Pathogen from SSI

Majority of isolates from SSI were resistant to regularly used antibacterials.* S. aureus* was resistant to ampicillin (71.4%), amoxicillin (66.7%), and trimethoprim-sulfamethoxazole (61.9%). Likewise, CoNS was resistant to ampicillin (84.6%), amoxicillin (84.6%), trimethoprim-sulfamethoxazole (61.5%), penicillin (61.5%), cloxacillin (38.5%), and cefoxidime (38.5%). In contrast, clindamycin (84.2%), cefoxitin (82.5%), cefixime (73.7%), and ceftriaxone (68.4%) were susceptible to Gram-positive isolates (Table 2).

On the other hand, Gram-negative isolates were resistant to ampicillin (88.6%), amoxicillin (79.5%), and trimethoprim-sulfamethoxazole (54.5%).* E. coli* was resistant to ampicillin (80%), amoxicillin (70%), and ceftriaxone (60%).* K. pneumoniae* was shown to be resistant to ampicillin (100%), amoxicillin (100%), trimethoprim-sulfamethoxazole (64.3%), ceftriaxone (57.1%), ceftazidime (28.6%), gentamycin (21.4%), and ciprofloxacin (14.3%).* Citrobacter *spp. were resistant to ampicillin (100%). In contrast, most Gram-negative isolates were susceptible to amikacin (95.5%) and ciprofloxacin (83.7%) (Table 3).

### 3.4. Multidrug Resistance Pattern of Bacterial Pathogens

Taking all bacterial isolates from SSI sample were shown 75% and 82.5% multidrug resistance (MDR = resistance of ≥2 drugs with different classes) for Gram negative and Gram positive bacteria respectively. Gram-negative bacteria such as* K. pneumoniae* were 12 (85.7%) resistant to ≥2 drugs in different class. Likewise, CoNS were 11 (84.6%) resistant to ≥2 drugs in different class (Table 4).

### 3.5. Characteristics of Risk Factors Attributed to Bacterial Infections

In bivariate logistic analysis, demographic and clinical factors such as age, occupation, level of education, gravidity, parity, prolonged labour, and history of diabetes mellitus were not significantly associated with bacterial infection in this study; however, mode of delivery and premature rapture of membrane were strongly associated in both bivariate and multivariate analyses for bacterial infection (*p* ≤ 0.05). Delivery by cesarean section and episiotomy were 102 and 86 times at risk for bacterial infection than instrumental deliveries (AOR (95% CI) = 102 (5.2, 2038, *p* = 0.002) and 86 (5, 1436, *p* = 0.002), resp.) (Table 5).

## 4. Discussion

Maternal infection during postnatal period was a vital factor for morbidity and mortality [3, 19]. In the current study, the possible etiologic agents causing morbidity among mothers during postnatal period were isolated. Three different biological wound specimens such as wound swab, aspirate, and biopsy were collected to ascertain possible pathogenic bacteria. From this study, the total prevalence of confirmed bacteria was 84.1%. This showed that women who delivered by cesarean section and episiotomy were vulnerable to various bacterial infections and this has continued even after delivery up to 30 days [1]. Considering the fact that postnatal women are part of vulnerable population and the effect could also influence the health status of the neonates, the current finding has shown high public health consequence. This is similar to studies conducted in Ethiopia and elsewhere [2, 20–22].

In the current study, the rate of pathogenic bacteria which were isolated from postnatal women was highly considerable for SSI. The predominant pathogens that cause SSIs are* S. aureus, E. coli, K. pneumoniae*, and CoNS. The reason for the predominance of those organisms may be the fact that most of the infected patients in this study had undergone cesarean section and episiotomy procedure for delivery which is favorable for different bacterial colonization commonly reported during SSI [3, 4]. This study was in line with studies conducted in Bahir Dar (83.3%) [9], Addis Ababa (84.1%) [3], and Nizwa Hospital, Oman (77.72%) [23]. But the current findings were higher compared to studies conducted in Gondar (31.5%) [24], Tanzania (61.8%) [22], Nepal (62.4%) [5], and Estonian University (6.2%) [7]. The possible explanation for this discrepancy might be due to poor wound care and aseptic technique during C/S and episiotomy procedure.

It is known that, from wound, there are different microbial contaminations. Wound cleaning without regular hand-washing antiseptics such as 70% alcohol and/or normal saline worsens the complications. To prevent further contamination, cleaning wounds with normal saline, covering with clean and dry bandage, giving patient education about personal hygiene, avoiding cross infection by restricting visitors, providing routine education regarding infection control measures, and following necessary SOP during sample collection should be applied [23]. The prevalence of pathogenic bacteria from wound was different, even though the study participants were similar. The difference might be related to accessibility of diagnostic materials, the use of aseptic technique during sample processing, and sample size. A study showed in Jimma [14] that the specimens were collected from women infected due to cesarean section for delivery including ovarian tumor operation and Myoma, but in the present study the specimens were collected from women infected due to cesarean section and episiotomy delivery.

Nowadays, emerging of high rate of bacteria resistant to multiple antibiotics is becoming a global threat by crossing worldwide borders and spreading between regions with significant speed. World health leaders have declared that drug-resistant organisms are frightening organisms that have a disastrous risk to persons in each country in the world [25]. Several factors augment this problem. Understanding bacterial profile and its antibiotic pattern is of paramount importance for proper management and minimizing the circulation of common pathogenic resistance strains in the community [13]. In this study, we tried to assess the sensitivity pattern of the isolated bacteria to different drugs in vitro. In the present finding,* S. aureus, E. coli, CoNS, *and* K. pneumoniae *are the commonest pathogens isolated from wound and were resistant to many antibiotics, indicating the big threat they poses. On the other hand, Gram-negative bacteria were susceptible to strong antibiotics like amikacin, ciprofloxacin, and others. This result is similar to studies in Bahir Dar [9], Addis Ababa [3], Mek'ele [26], and Palestine [19]. However, as long as there is high rate of irrational use of antibiotics as well as poor adherence in the society, these antibiotics may no longer be effective to pathogens. Different antibacterial susceptibility patterns were observed for bacterial isolates collected from wound sites. One of the commonest isolated bacteria from wound site was* S. aureus*, which was resistant to ampicillin (71.4%), amoxicillin (66.7%), and trimethoprim-sulfamethoxazole (61.9%). More or less this is in agreement with a study done in Bahir Dar [9]. Similarly, CoNS was highly resistant to ampicillin (84.6%), amoxicillin (84.6%), and trimethoprim-sulfamethoxazole (63.6%). This is in agreement with a study done in Bahir Dar [9]. Among Gram-negative bacteria,* E. coli* was highly resistant to ampicillin (80%), amoxicillin (70%), and ceftriaxone (60%). This finding is in line with a study done in Addis Ababa [3, 23]. In addition,* K. pneumoniae *was shown to be fully resistant to amoxicillin (100%) and ampicillin (100%) and strongly resistant to trimethoprim-sulfamethoxazole (64.3%) and ceftriaxone (57.1%). This is in agreement with a study done in Addis Ababa and Debre Markos [3, 25].

In the current study, multidrug resistance (MDR) bacterial isolate showed 75% and 82.5% resistance to Gram-negative and Gram-positive bacteria, respectively; this is in line with a study done in Addis Ababa [3]. Several reasons could have supported such level of resistance; this includes mismanagement of drugs by health specialists, unexperienced experts, and untrained individuals. In addition to this, most drugs in the study area can be bought deprived of laboratory confirmation, which leads to misuse of drugs by the community and thus causes the occurrence and spread of antibacterial tolerance. Additional fundamental reason could be reduced hospital sanitation, contributing to the spread of those drug-resistant bacteria in the area [24, 25]. This situation raises serious concerns. Moreover, the development of high resistance gene pool may increase antibacterial resistance. Taken together, these findings clearly show how resistance strains are expanding at an alarming rate in the area. With this trend, an antibiotic that was effective a year ago might no longer be used. This creates great burden, especially to people living in resource-poor countries, where they could not ensure their daily bread let alone for medication. The cost of new antibiotics is also high, which in turn poses great burden for poor countries [27].

In addition to the identification of the common bacteria and their resistance pattern, this study tried to evaluate the association of different sociodemographic and clinical parameters with bacterial infection. In the present study, the prevalence of bacteria was associated with obstetric parameters like mode of delivery through cesarean section and vaginal delivery by using episiotomy (*p* = 0.002). This could be attributed to the physiological change, immune shift, high bleeding, and contamination; this is in line with studies conducted in Bahir Dar [9], Jimma [4, 14], and Estonian University [10]. There was also an association of developing bacterial infection with premature rapture of membrane (*p* = 0.018). This is in line with studies conducted in Jimma [4], Tanzania [11], Hong Kong [28], and Brazil [29]. This might be due to the fact that having a premature rapture of membrane could expose women to various injuries, which again enable pathogenic bacteria to have suitable environment to cause infection. The presence of wound infection following cesarean section and episiotomy was aggravated due to improper wound care and improper treatment. In addition, women who have got premature rapture of membrane could get exposed to various injuries and minor tears, which again enable pathogenic bacteria to have convenient environment to cause infection.

The limitation of anaerobic culture facility that is used to cultivate anaerobic bacteria might increase the quantity of bacterial identification. Since it is a cross-sectional study, it has limited capacity to assess risk factors.

## 5. Conclusion and Recommendations

The prevalence of bacterial infection was too increased among women who attended postnatal care. The major bacteria were* S. aureus*,* E. coli, *CoNS*, K. pneumoniae*,* Citrobacter*,* Enterobacter*, and* S. pyogenes*. Gram-negative and Gram-positive bacteria revealed resistance to the most frequently used drugs tested in vitro. Multidrug-resistant bacteria show big threat posed by antibiotic resistant strains in vulnerable mothers. Drug-resistant bacterial infection leads to increased patient hospital stay, health care costs, and death rate. Factors such as cesarean section, episiotomy for delivery, and premature rapture of membrane were predictors for bacterial infection among postnatal mothers. Reviewing the nature and cause of bacterial infection and its drug resistance is necessary to overcome bacterial infection. To confirm applicable treatment, the existing information about bacteria that cause maternal infections and their drug resistance pattern is essential.

## Figures and Tables

**Table 1:** Sociodemographic, obstetric, and clinical variables from women attending antenatal and postnatal services at the University of Gondar Teaching Hospital from 1 January 2016 to 30 May 2016.

Characteristics	Number (%)
Age (years)	
15–24	39 (36.5%)
25–34	56 (52.3%)
35–44	12 (11.2%)
Residence	
Urban	83 (77.6%)
Rural	24 (23.4%)
Religion	
Orthodox	97 (90.7%)
Muslim	7 (6.5%)
Protestant	3 (2.8%)
Marital status	
Married	105 (98.1)
Single	2 (1.9%)
Educational level	
Illiterate	35 (32.7%)
Primary school (1–8)	14 (13.1%)
Secondary school	31 (29.0%)
Higher education	27 (25.2%)
Occupational status	
Housewives	65 (60.7%)
Self-employees	17 (15.9%)
Government employees	20 (18.7%)
Students	3 (2.8%)
Daily laborer	2 (1.9%)
Gravidity	
1–3	95 (88.8%)
4–6	10 (9.3%)
7–9	2 (1.9%)
Prolonged labour	
Yes	52 (48.6)
No	55 (51.4%)
Premature rapture of membrane	
Yes	43 (40.2%)
No	64 (59.8%)
Mode of delivery	
Vaginal delivery	69 (41.3%)
Cesarean delivery	81 (48.5%)
Instrumental delivery	17 (10.2%)
Place of delivery	
Health institution	107 (100%)
Diabetes mellitus	
No	98 (91.6%)
Yes	9 (8.4%)
HIV/AIDS	
No	103 (96.3%)
Yes	4 (3.7%)

HIV: human immunodeficiency virus.

**Table 2:** Antibacterial susceptibility pattern for Gram-positive SSI causing pathogens from women attending postnatal service at University of Gondar Teaching Hospital from 1 January 2016 to 30 May 2016.

Bacterial isolates		Antibacterial susceptibility pattern									
		AMP	AMO	CRO	P	CAZ	SXT	CXM	CXC	DA	CXT
*S. aureus* (*n* = 42)	S%	12 (28.6)	14 (33.3)	27 (64.3)	21 (50)	24 (57.1)	16 (38.1)	31 (73.8)	20 (47.6)	35 (83.3)	35 (83.3)
	R%	30 (71.4)	28 (66.7)	15 (35.7)	21 (50)	18 (42.9)	26 (61.9)	11 (26.2)	22 (52.4)	7 (16.7)	7 (16.7)
*CoNS* (*n* = 13)	S%	2 (15.4)	2 (14.3)	8 (61.5)	5 (38.5)	8 (61.5)	5 (38.5)	10 (76.9)	8 (61.5)	12 (92.3)	11 (84.6)
	R%	11 (84.6)	11 (85.7)	5 (38.5)	8 (61.5)	5 (38.5)	8 (61.5)	3 (23.1)	5 (38.5)	1 (7.7)	2 (15.4)
*S. pyogenes* (*n* = 2)	S%	0 (0)	1 (50)	4 (100)	2 (100)	1 (50)	1 (50)	1 (50)	1 (50)	1 (50)	1 (50)
	R%	2 (100)	1 (50)	0 (0)	0 (0)	1 (50)	1 (50)	1 (50)	1 (50)	1 (50)	1 (50)
*Total *(*n* = 57)	*S%*	*14 (24.6)*	*17 (29.8)*	*39 (68.4)*	*28 (49.1)*	*33 (57.9)*	*22 (38.6)*	*42 (73.7)*	*29 (50.9)*	*48 (84.2)*	*47 (82.5)*
	*R%*	*43 (75.4)*	*40 (70.2)*	*18 (31.6)*	*29 (50.9)*	*24 (42.1)*	*35 (61.4)*	*15 (26.3)*	*28 (49.1)*	*9 (15.8)*	*10 (17.5)*

P: penicillin; CAZ: ceftazidime; CXM: cefixime; CXC: cloxacillin; CD: clindamycin; CXT: cefoxitin; *S. pyogenes: Streptococcus pyogenes.*

**Table 3:** Antibacterial susceptibility pattern for Gram-negative SSI causing pathogens from women attending postnatal service at University of Gondar Teaching Hospital from 1 January 2016 to 30 May 2016.

Bacterial isolates		Antibacterial susceptibility pattern											
		AMX	AMP	CRO	CIP	CXT	CAZ	SXT	CXM	GEN	NA	AMK	TTC
*E. coli *(*n* = 20)	S%	6 (30)	4 (20)	8 (40)	16 (80)	16 (80)	12 (60)	10 (50)	15 (75)	13 (65)	15 (75)	19 (95)	6 (30)
	R%	14 (70)	16 (80)	12 (60)	4 (20)	4 (20)	8 (40)	10 (50)	5 (25)	7 (35)	5 (25)	1 (5)	14 (70)
*K. pneumoniae *(*n* = 14)	S%	0 (0)	0 (0)	6 (42.9)	12 (85.7)	13 (92.9)	10 (71.4)	5 (35.7)	11 (78.6)	11 (78.6)	13 (92.9)	14 (100)	2 (14.3)
	R%	14 (100)	14 (100)	8 (57.1)	2 (14.3)	1 (7.1)	4 (28.6)	9 (64.3)	3 (21.4)	3 (21.4)	1 (7.1)	0 (0)	12 (86)
*Citrobacter *spp.(*n* = 4)	S%	2 (50)	0 (0)	4 (100)	4 (100)	2 (50)	2 (50)	3 (75)	2 (50)	2 (50)	3 (75)	3 (75)	2 (50)
	R%	2 (50)	4 (100)	0 (0)	0 (0)	2 (50)	2 (50)	1 (25)	2 (50)	2 (50)	1 (25)	1 (25)	2 (50)
*Enterobacter *spp.(*n* = 4) *P. aeruginosa *(*n* = 1)	S%	1 (25)	1 (25)	3 (25)	3 (75)	4 (100)	3 (75)	2 (50)	4 (100)	3 (75)	3 (75)	4 (100)	2 (50)
	R%	3 (75)	3 (75)	1 (25)	1 (25)	0 (0)	1 (25)	2 (50)	0 (0)	1 (25)	1 (25)	0 (0)	2 (50)
	S%	0 (0)	0 (0)	1 (100)	1 (100)	1 (100)	1 (100)	0 (0)	1 (100)	0 (0)	1 (100)	1 (100)	0 (0)
	R%	1 (100)	1 (100)	0 (0)	0 (0)	0 (0)	0 (0)	1 (100)	0 (0)	1 (100)	0 (0)	(0)	1 (100)
*P. mirabilis *(*n* = 1)	S%	0 (0)	0 (0)	1 (100)	1 (100)	0 (0)	0 (0)	0 (0)	0 (0)	0 (0)	1 (100)	1 (100)	0 (0)
	R%	1 (100)	1 (100)	0 (0)	0 (0)	1 (100)	1 (100)	1 (100)	1 (100)	1 (100)	0 (0)	0 (0)	1 (100)
*Total = 44*	*S%*	*9 (20.5)*	*5 (11.4)*	*23 (52.3)*	*37 (83.7)*	*36 (80.1)*	*28 (63.6)*	*20 (45.5)*	*33 (75)*	*29 (65.9)*	*36 (81.8)*	*42 (95.5)*	*12 (27.3)*
	*R%*	*35 (79.5)*	*39 (88.6)*	*21 (47.7)*	*7 (16.3)*	*8 (19.9)*	*16 (36.4)*	*24 (54.5)*	*11 (25)*	*15 (34.1)*	*8 (18.2)*	*2 (4.5)*	*32 (72.7)*

CIP: ciprofloxacin; GEN: gentamycin; AMK: amikacin; NA: nalidixic acid; TTC: tetracycline; SXT: sulfamethoxazole trimethoprim; R: resistance; S: sensitivity.

**(a):** 

Antibiograms	Gram-negative bacterial isolates						
		*E. coli*	*K. pneumoniae*	*Citrobacter *spp.	*Enterobacter *spp.	*P. mirabilis*	*P. aeruginosa*
	Total = *44*	= 20	= 14	= (4)	= (4)	= 1	* = 1*
AMP, TTC	5 (11.4)	2 (10)	3 (21.4)	—	—	—	—
AMO, SXT	1 (2.3)	—	1 (11.1)	—	—	—	—
AMP, TTC, GEN	1 (2.3)	1 (5)	—	—	—	—	—
AMP, TTC, SXT	3 (6.8)	1 (5)	2 (14.3)	—	—	—	—
AMO, SXT, CRO	1 (2.3)	1 (5)	—	—	—	—	—
AMX, TTC, CRO	1 (2.3)	1 (5)	—	—	—	—	—
AMX, TTC, CRO	1 (2.3)	1 (5)	—	—	—	—	—
AMX, CRO, GEN, TTC	1 (2.3)	1 (5)	—	—	—	—	—
AMX, TTC, SXT, CRO	5 (11.4)	1 (5)	2 (14.3)	—	1 (25)	—	1 (100)
AMP, SXT, CRO, NAL	2 (4.5)	2 (10)	—	—	—	—	—
AMO, SXT, CXM, TTC	1 (2.3)	—	—	1 (25)	—	—	—
AMO, CPR, CRO, NAL	1 (2.3)	1 (5)	—	—	—	—	—
AMO, TTC, SXT, GEN, CRO	4 (9.1)	1 (5)	3 (21.4)	—	—	—	—
AMO, CXM, AMK, TTC, NAL	1 (2.3)	—	—	1 (25)	—	—	—
AMO, SXT, CXM, GEN, TTC	1 (2.3)	—	—	—	—	1 (100)	—
AMO, CRO, CPR, SXT, TTC, NAL	1 (2.3)	1 (5)	—	—	—	—	—
AMO, CPR, SXT, CXM, TTC, NAL	1 (2.3)	—	1 (11.1)	—	—	—	—
AMO, CRO, CPR, SXT, GEN, TTC, NAL	3 (6.8)	2 (10)	—	—	1 (25)	—	—

*Total *	*33 (75)*	*15 (75)*	*12 (85.7)*	*2 (50)*	*2 (50)*	*1 (100)*	*1 (100)*

**(b):** 

Antibiograms	Gram-positive bacterial isolates			
	Total = 57	*S. aureus* = 42	CoNS = 13	*S. pyogenes* = 2
P, CXM	1 (1.8)	1 (2.4)	—	—
P, CRO	1 (1.8)	—	1 (7.7)	—
AMP, CXC	2 (3.5)	1 (2.4)	1 (7.7)	—
AMP, CAZ	2 (3.5)	1 (4.8)	1 (7.7)	—
AMO, SXT	6 (10.5)	5 (11.9)	1 (7.7)	—
P, CRO, SXT	1 (1.8)	1 (2.4)	—	—
AMP, SXT, CAZ	3 (5.3)	1 (2.4)	2 (15.4)	—
AMO, CAZ, CXC	3 (5.3)	3 (7.1)	—	—
AMO, CRO, CXC	4 (7)	4 (9.5)	—	—
AMO, CRO, SXT	2 (3.5)	2 (4.8)	—	—
AMP, SXT, CXC	2 (3.5)	1 (2.4)	1 (7.7)	—
P, SXT, CXC, CD	2 (3.5)	2 (4.8)	—	—
P, CRO, SXT, CXC	6 (10.5)	4 (9.5)	2 (15.4)	—
AMP, CAZ, SXT, CXC	4 (7)	2 (4.8)	1 (7.7)	1 (50)
AMP, CRO, CXC, SXT	3 (5.3)	3 (7.1)	—	—
P, CRO, SXT, CXC, DA	1 (1.8)	1 (2.4)	—	—
P, SXT, CAZ, CXC, DA	2 (3.5)	1 (2.4)	1 (7.7)	—
AMO, CRO, SXT, CXC, DA	2 (3.5)	2 (4.8)	—	—

*Total*	*47 (82.5)*	*35 (83.3)*	*11 (84.6)*	*1 (50)*

TTC: tetracycline; GEN: gentamycin; NA: nalidixic acid; CPR: ciprofloxacin; CoNS: coagulase negative *Staphylococcus* species; GAS: group A *Streptococcuspyogenes*; CD: clindamycin; CXC: cloxacillin.

**Table 5:** Bivariate and multivariate analysis for the assessment of factors associated with bacterial infection from women attending antenatal and postnatal service at the University of Gondar Teaching Hospital from 1 January 2016 to 30 May 2016.

Characteristics	Culture		Bivariate analysis	Multivariate analysis	*p* value
	Positive number (%)	Negative number (%)	COR (95% CI)	AOR (95% CI)	
Age					
15–24	34 (37.8)	5 (29.4)	1		
25–34	49 (54,4)	7 ( 41.2)	2.267 (0.454, 11.327)		
35–44	7 (7.8)	5 (29.4)	1.741 (0.393, 7.713)		
Residence					
Urban	70 (77.8)	13 (76.5)	0.923 (0.273, 3.164)		
Rural	20 (22.2)	4 (23.5)	1		
Occupation					
Self-employer	85 (94,4)	17 (100)	1		
Government	5 (5.6)	0 (0)	0.982 (0.585, 1.648)		
Education					
Illiterate	28 (31.1)	7 (41.2)	1		
Primary	12 (13.3)	2 (11.8)	0.696 (0.181, 2.674)		
Secondary	27 (30)	4 (23.5)	1.043 (0.167, 6.59)		
Higher education	23 (25.6)	4 (23.5)	1.174 (0.264, 5.226)		
Marital status					
Married	88 (97.8)	17 (100)	1		
Single	2 (2.2)	0 (0)	0.691 (0.227, 2.101)		
Mode of delivery					
VDE	34 (37.8)	3 (17.6)	15.111 (3.095, 73.77)	102 (5.2, 2028)	0.002
C/S	50 (55.6)	6 (35.3)	11.111 (2.865, 43.099)	86 (5, 1436)	0.002
Instrumental	6 (6.7)	8 (47.1)	1		
Prolonged labour					
No	45 (50)	10 (58.8)	1		
Yes	45 (50)	7 (41.2)	0.700 (0.245, 2.001)		
PRM					
No	50 (55.6)	14 (82.4)	1		
Yes	40 (44.4)	3 (17.6)	0.268 (0.072, 0.997)	0.012 (0.001, 0.305)	0.008
Gravidity					
1–3	80 (88.9)	15 (88.2)	1.746 (0.513, 5.940)		
4–6	9 (10)	1 (5.9)	1.290 (0.343, 4.856)		
7–9	1 (1.1)	1 (5.9)	1		

*Note*. *∗*  indicates  statistical significance at *p* ≤ 0.05, in both bivariate and multivariate analyses. AOR: adjusted odds ratio; COR: crude odds ratio; 1: reference group; 95% CI: 95% confidence interval; UTI: urinary tract infection; C/S: cesarean section; VDE: vaginal delivery with episiotomy; PRM: premature rapture of membrane.
